# Gadolinium-enhanced intracranial aneurysm wall imaging and risk of aneurysm growth and rupture: a multicentre longitudinal cohort study

**DOI:** 10.1007/s00330-023-10388-7

**Published:** 2023-12-18

**Authors:** Laura T. van der Kamp, Myriam Edjlali, Olivier Naggara, Toshinori Matsushige, Diederik O. Bulters, Ronneil Digpal, Chengcheng Zhu, David Saloner, Peng Hu, Xiaodong Zhai, Mahmud Mossa-Basha, Bing Tian, Shigeyuki Sakamoto, Qichang Fu, Ynte M. Ruigrok, Huilin Zhao, Huijun Chen, Gabriel J. E. Rinkel, Irene C. van der Schaaf, Mervyn D. I. Vergouwen

**Affiliations:** 1 Department of Neurology and Neurosurgery, UMC Utrecht Brain Center, Utrecht University, University Medical Center Utrecht, room number G3-201, Postbox 85500, 3508 Utrecht, GA The Netherlands; 2 Department of Radiology, APHP, Hôpitaux Raymond-Poincaré and Ambroise Paré, DMU Smart Imaging, Laboratoire d’imagerie Biomédicale Multimodale (BioMaps), GH Université Paris-Saclay, Université Paris-Saclay, CEA, CNRS, Inserm, Service Hospitalier Frédéric Joliot, Orsay, France; 3 Department of Neuroradiology, Université de Paris, IMABRAIN-INSERM-UMR1266, DHU-Neurovasc, GHU Paris, Centre Hospitalier Sainte-Anne, Paris, France; 4 Department of Neurosurgery and Interventional Neuroradiology, Hiroshima City Asa Citizens Hospital, Hiroshima, Japan; 5 Department of Neurosurgery, University Hospital Southampton, University Hospital Southampton NHS Foundation Trust, Southampton, UK; 6 Department of Radiology, University of Washington School of Medicine, Seattle, WA USA; 7 Department of Radiology and Biomedical Imaging, University of California San Francisco, San Francisco, CA USA; 8 Department of Neurosurgery, Capital Medical University Xuanwu Hospital, Capital Medical University, Bejing, China; 9 Department of Radiology, University of North Carolina, Chapel Hill, NC USA; 10 Department of Radiology, Changhai Hospital, Shanghai, China; 11 Department of Neurosurgery and Interventional Neuroradiology, Graduate School of Biomedical and Health Sciences, Hiroshima University, Hiroshima, Japan; 12 Department of Magnetic Resonance, The First Affiliated Hospital of Zhengzhou University, Zhengzhou, China; 13 Department of Radiology, Ren Ji Hospital, Shanghai Jiao Tong University School of Medicine, Shanghai, China; 14 Department of Biomedical Engineering, School of Medicine, Tsinghua University, Bejing, China; 15 Department of Radiology, UMC Utrecht Brain Center, University Medical Center Utrecht, Utrecht University, Utrecht, The Netherlands

**Keywords:** Brain, Intracranial aneurysm, Magnetic resonance imaging, Risk factors

## Abstract

**Objectives:**

In patients with an unruptured intracranial aneurysm, gadolinium enhancement of the aneurysm wall is associated with growth and rupture. However, most previous studies did not have a longitudinal design and did not adjust for aneurysm size, which is the main predictor of aneurysm instability and the most important determinant of wall enhancement. We investigated whether aneurysm wall enhancement predicts aneurysm growth and rupture during follow-up and whether the predictive value was independent of aneurysm size.

**Materials and methods:**

In this multicentre longitudinal cohort study, individual patient data were obtained from twelve international cohorts. Inclusion criteria were as follows: 18 years or older with ≥ 1 untreated unruptured intracranial aneurysm < 15 mm; gadolinium-enhanced aneurysm wall imaging and MRA at baseline; and MRA or rupture during follow-up. Patients were included between November 2012 and November 2019. We calculated crude hazard ratios with 95%CI of aneurysm wall enhancement for growth (≥ 1 mm increase) or rupture and adjusted for aneurysm size.

**Results:**

In 455 patients (mean age (SD), 60 (13) years; 323 (71%) women) with 559 aneurysms, growth or rupture occurred in 13/194 (6.7%) aneurysms with wall enhancement and in 9/365 (2.5%) aneurysms without enhancement (crude hazard ratio 3.1 [95%CI: 1.3–7.4], adjusted hazard ratio 1.4 [95%CI: 0.5–3.7]) with a median follow-up duration of 1.2 years.

**Conclusions:**

Gadolinium enhancement of the aneurysm wall predicts aneurysm growth or rupture during short-term follow-up, but not independent of aneurysm size.

**Clinical relevance statement:**

Gadolinium-enhanced aneurysm wall imaging is not recommended for short-term prediction of growth and rupture, since it appears to have no additional value to conventional predictors.

**Graphical abstract:**

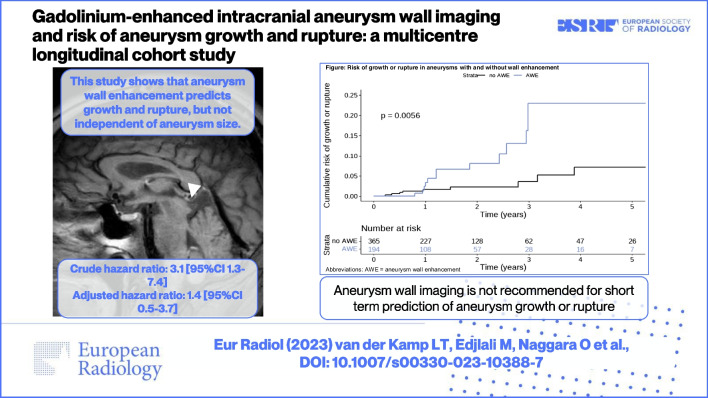

**Key Points:**

*• Although aneurysm wall enhancement is associated with aneurysm instability in cross-sectional studies, it remains unknown whether it predicts risk of aneurysm growth or rupture in longitudinal studies.*

*• Gadolinium enhancement of the aneurysm wall predicts aneurysm growth or rupture during short-term follow-up, but not when adjusting for aneurysm size.*

*• While gadolinium-enhanced aneurysm wall imaging is not recommended for short-term prediction of growth and rupture, it may hold potential for aneurysms smaller than 7 mm.*

**Supplementary Information:**

The online version contains supplementary material available at 10.1007/s00330-023-10388-7.

## Introduction

In a patient with a newly diagnosed unruptured intracranial aneurysm (UIA), the risk of aneurysm rupture needs to be weighed against the risk of treatment complications from preventive endovascular or neurosurgical aneurysm treatment [1, 2]. Important predictors for aneurysm rupture are size, site, and shape of the aneurysm, family history, smoking, and hypertension [1, 3–6]. Size is a consistent predictor and is included in scores predicting future rupture risk, with smaller intracranial aneurysms having a lower risk of rupture [1, 3]. Paradoxically, most episodes of aneurysmal subarachnoid haemorrhage (SAH) result from rupture of small aneurysms. This is partially explained by the higher prevalence of small aneurysms [7]. To better advise patients with a small aneurysm, it is important to identify the subset of those patients who have an increased risk of growth or rupture.

Gadolinium-enhanced MRI aneurysm wall imaging is a promising technique to improve risk prediction in UIAs. After administration of gadolinium, enhancement of the aneurysm wall may occur, which is thought to represent wall inflammation, slow flow, or wall permeability [8–15]. Because aneurysm wall enhancement occurs in almost all recently ruptured aneurysms compared to approximately 30% of small unruptured aneurysms, it may be a novel biomarker to identify unstable aneurysms [16]. Since most previous studies had a cross-sectional design, comparing ruptured with unruptured aneurysms or comparing unstable with stable aneurysms, the higher proportion of enhancement in ruptured or growing aneurysms could be the result of either the growth or rupture, or a risk factor for growth or rupture [17, 18]. Therefore, it remains unclear whether aneurysm wall enhancement predicts future growth or rupture. In addition, previous studies did not adjust for aneurysm size. Larger aneurysms, with an inherent increased risk of growth or rupture, more often show aneurysm wall enhancement than smaller aneurysms [16, 19–24]. Thus, to assess whether wall enhancement is an independent predictor of growth or rupture, it is necessary to adjust for aneurysm size in the analysis.

Our primary aim was to investigate if aneurysm wall enhancement predicts aneurysm growth or rupture during follow-up in a large international cohort of patients with a UIA < 15 mm. Our secondary aim was to investigate if wall enhancement predicts aneurysm growth or rupture independent of aneurysm size.

## Methods

### Ethics

The protocol of the study was approved by the Institutional Review Board. This analysis of existing data from cohorts of patients did not require formal approval from an ethics committee, according to the accredited Medical Research Ethics Committee. The individual institutions received approval for retrospective review of the imaging and clinical data, with waiver of consent because data were de-identified.

This study followed the Strengthening the Reporting of Observational Studies in Epidemiology (STROBE) reporting guideline.

### Participants

Individual patient and aneurysm data were retrospectively obtained of patients with at least one saccular UIA who had baseline aneurysm wall imaging and MRA between November 2012 and November 2019 from twelve cohorts of the following countries: the USA (two centres), UK (one centre), The Netherlands (one centre), France (one centre), China (five centres), and Japan (two centres). Inclusion criteria were as follows: (1) 18 years or older with one or more untreated unruptured intracranial aneurysms; (2) gadolinium-enhanced MRI aneurysm wall imaging and MRA at baseline; and (3) MRA or rupture during follow-up. Exclusion criteria were as follows: (1) extradural aneurysm; (2) fusiform aneurysm; and (3) arteriovenous malformation-related aneurysm. We also excluded aneurysms ≥ 15 mm since these aneurysms have a high risk of rupture and therefore usually undergo preventive endovascular or neurosurgical treatment. As a result, the research question of the current study has limited value in that subgroup of aneurysms. We used individual patient data from unpublished and published cohorts (eTable 1) [15, 25, 26]. The data of the published cohorts were updated for the purpose of this study and patients who did not fulfil the inclusion criteria were excluded.

### Measurements

The local investigators collected the following data from medical records: patients’ age, sex, population (Japanese, or other), history of hypertension (yes/no), earlier SAH (yes/no), current smoking (yes/no), family history of SAH (yes/no), date of first aneurysm wall imaging, field strength of aneurysm wall imaging (1.5, 3, or 7 T), date of last follow-up MRA, aneurysm growth during follow-up (yes/no), date of MRA that observed growth, date of last MRA before growth, aneurysm rupture during follow-up (yes/no), date of aneurysm rupture, and follow-up duration in patients without growth or rupture (until last MRA).

### Imaging

All patients had gadolinium-enhanced MRI aneurysm wall imaging and MRA at baseline. The technique parameters are described in eTable 2. The local neuroradiologist compared the pre- and post-contrast imaging, to assess the presence of wall enhancement (yes/no), irrespective of pattern of enhancement. The follow-up MRA was performed according to the local protocol for the timing of follow-up imaging. The local neuroradiologist recorded the aneurysm size (maximum diameter on a 0.1-mm scale), aneurysm site, and aneurysm irregularity (yes/no) at baseline and follow-up imaging. Irregular aneurysm shape was defined as the presence of blebs, aneurysm wall protrusions, daughter sacs, or multiple lobes [27]. For patients with two or more intracranial aneurysms, each aneurysm was evaluated separately.

### Outcome assessment

The primary outcome was aneurysm growth or rupture during follow-up. Aneurysm growth was defined as a ≥ 1 mm increase in at least one direction [28].

### Power calculation

A Cox regression of the log hazard ratio (HR) on a covariate with a standard deviation of 0.48 based on a fixed number of 559 aneurysms was used to detect a regression coefficient equal to 1.0986, which corresponds with a HR of 3.0. The calculation was adjusted since a multiple regression of the variable of interest, aneurysm wall enhancement, on the other covariate, aneurysm size, in the Cox regression is expected to have an R-squared of 0.20 [8, 19–22]. Assuming a risk of aneurysm growth or rupture of 10%, 94% power will be achieved at a 0.05 significance level.

### Statistical analyses

A descriptive analysis of the studied population was performed. All continuous variables were checked for normality. The analyses were aneurysm-based since our determinant, aneurysm wall enhancement (AWE), and our outcomes, growth and rupture, were all aneurysm-specific. In our primary analysis, we investigated the risk of aneurysm growth or rupture during follow-up of aneurysms with and without gadolinium enhancement of the aneurysm wall at baseline. The risk difference between the two groups was determined with a 95%CI with the Clopper-Pearson test. A Cox proportional hazard model was used to determine the HR of aneurysm growth or rupture during follow-up in aneurysms with and without wall enhancement. The assumptions of proportional hazard were tested. A stratified analysis was performed to investigate potential sex differences in the risk of growth or rupture. In our secondary analysis, the HR of wall enhancement on aneurysm growth or rupture was calculated when adjusting for aneurysm size. In this bivariable model, aneurysm size was analysed as a continuous variable. To examine the absolute risk of growth or rupture for aneurysms with and without aneurysm wall enhancement, the Kaplan-Meier estimate was used, and the hazard function was visualised with a Kaplan-Meier curve.

Furthermore, we performed a subgroup analysis for small aneurysms, defined as aneurysms with a diameter < 7 mm. These aneurysms have a low risk of rupture and therefore usually do not undergo preventive endovascular or neurosurgical treatment. Instead, they are often monitored radiologically. Nevertheless, because < 7 mm aneurysms are much more prevalent than larger aneurysms, most instances of subarachnoid haemorrhage result from rupture of an aneurysm < 7 mm. In this subgroup analysis, we calculated both unadjusted and adjusted hazard ratios. The log-rank test pooled over strata was used to test the equality of the survival distributions of the different levels. A *p* value less than .05 indicated statistical significance.

Finally, we performed a subgroup analysis for patients who had 3T aneurysm wall imaging, excluding those who were scanned on another field strength (1.5T or 7T). In this subgroup analysis, we calculated unadjusted and adjusted hazard ratios.

Four variables (history of hypertension, earlier SAH, current smoking, and family history of SAH) showed missing values. As we did not include these variables in the analysis to answer the primary and secondary research questions, the final analyses were based on complete data. Statistical analyses were performed in R version 4.0.2 (The R Foundation) using the packages car (Fox J, Weisberg S (2002)), dplyr (Wickham H (2014)), ggplot2 (Wickham H (2007)), survival (Therneau TM, Grambsch PM (1997)), and survminer (Kassambara A (2017)).

## Results

We included 455 patients with 559 aneurysms. Patient and aneurysm characteristics are shown in Table 1. The mean age of the included patients was 60 years, and 323 patients (71%) were female. The total follow-up duration was 994 aneurysm-years (median 1.2 years [IQR 0.6–2.4]). Aneurysm wall enhancement was observed in 194 (35%) aneurysms. The median size of aneurysms with wall enhancement at baseline was 5.6 mm compared to 3.5 mm for aneurysms without wall enhancement. Of the 81 patients with multiple aneurysms (2 aneurysms: *n*= 66; 3 aneurysms: *n*= 11; 4 aneurysms: *n*= 4), 44 patients had at least 1 aneurysm with wall enhancement.

**Table 1:** Descriptive data for baseline stratified to wall enhancement

			Wall enhancement at baseline	
Patient characteristics	All (*N *= 455)	*N*, missing	Present (*N *= 168)	Absent (*N *= 287)
Female sex	323 (71%)	0	127 (76%)	196 (68%)
Mean age, years (SD)	60 (13)	0	63 (12)	59 (13)
Japanese	99 (22%)	0	29 (17%)	70 (24%)
Previous SAH from another aneurysm	48 (11%)	8	11 (7%)	37 (13%)
Hypertension	201 (44%)	4	78 (46%)	123 (43%)
Current smoker	110 (24%)	6	36 (21%)	74 (26%)
Family history of SAH	26 (6%)	8	9 (5%)	17 (6%)
Aneurysm characteristics	All (*N *= 559)	*N*, missing	Present (*N *= 194)	Absent (*N *= 365)
Site				
ICA	174 (31%)	0	46 (24%)	128 (35%)
MCA	209 (37%)	0	81 (42%)	128 (35%)
Acom	76 (14%)	0	28 (14%)	48 (13%)
Pcom	33 (6%)	0	11 (6%)	22 (6%)
Vertebrobasilar arteries (excluding basilar tip)	28 (5%)	0	11 (6%)	17 (5%)
ACA	24 (4%)	0	11 (6%)	13 (4%)
Basilar tip	15 (3%)	0	6 (3%)	9 (3%)
Median size, mm (IQR)	4 (3–6)	0	6 (4–7)	4 (3–4)
Irregular shape	145 (26%)	0	78 (40%)	67 (18%)
Field strength				
1.5T	115 (21%)	0	32 (16%)	83 (23%)
3T	443 (79%)	0	161 (83%)	282 (77%)
7T	1 (0%)	0	1 (1%)	0 (0%)
Median follow-up duration, years (IQR)	1.2 (0.6–2.4)	0	1.1 (0.5–2.2)	1.3 (0.7–2.5)

Abbreviations: *SD* standard deviation, *SAH* subarachnoid haemorrhage, *ICA* internal carotid artery, *MCA* middle cerebral artery, *Pcom* posterior communicating artery, *Acom* anterior communicating artery, *IQR* interquartile range

### Risk of growth and rupture in aneurysms with and without wall enhancement

During follow-up, 21 patients had seventeen growing aneurysms without rupture (10/17 had wall enhancement) and five ruptured aneurysms (3/5 had wall enhancement) (Table 2). Two of the 21 patients with aneurysm growth or rupture had multiple aneurysms. One patient had four aneurysms; the two largest aneurysms (12 mm middle cerebral artery (MCA) and 9 mm anterior cerebral artery aneurysm) had AWE at baseline and grew during follow-up, while the two smaller aneurysms (4 mm anterior communicating artery and 2 mm MCA aneurysm) had no AWE at baseline and did not grow during follow-up. The other patient had three aneurysms (4 mm, 3 mm, and 2 mm internal carotid artery aneurysms), of which only the smallest showed AWE at baseline and grew during follow-up.

**Table 2:** Descriptive data for stable and unstable aneurysms during follow-up

Baseline characteristics	Primary outcome	
	Stable during FU	Growth or rupture during FU
Patient characteristics	*N* = 435	*N* = 20
Female sex	308 (71%)	15 (75%)
Mean age (SD)	60 (13)	65 (13)
Japanese	94 (23%)	5 (25%)
Previous SAH from another aneurysm †	47 (11%)	1 (5%)
Hypertension ‡	189 (43%)	12 (60%)
Current smoker §	104 (24%)	6 (30%)
Family history of SAH ∥	26 (6%)	0 (0%)
Aneurysm characteristics	*N* = 537	*N* = 22
Site		
ICA	168 (31%)	6 (27%)
MCA	203 (38%)	6 (27%)
Pcom	32 (6%)	1 (5%)
Acom	73 (14%)	3 (14%)
ACA	21 (4%)	3 (14%)
Basilar tip	13 (2%)	2 (9%)
Vertebrobasilar arteries	27 (5%)	1 (5%)
Median size, mm (IQR)	4 (3–6)	5.5 (4–10.5)
Irregular shape	137 (26%)	8 (36%)
Aneurysm wall enhancement	181 (34%)	13 (59%)
Mean PHASES score (SD)	4 (3)	6 (5)
Median follow-up duration, years (IQR)	1.2 (0.6–2.3)	1.1 (0.7–1.6)

^†^ 8 missing values, ‡ 4 missing values, § 6 missing values, ∥ 8 missing values. Abbreviations: *FU* follow-up, *SD* standard deviation, *SAH* subarachnoid haemorrhage, *ICA* internal carotid artery, *MCA* middle cerebral artery, *Pcom* posterior communicating artery, *Acom* anterior communicating artery, *IQR* interquartile range, PHASES Population, Hypertension, Age, Size, Earlier subarachnoid haemorrhage, and Site

**Fig. 1:**
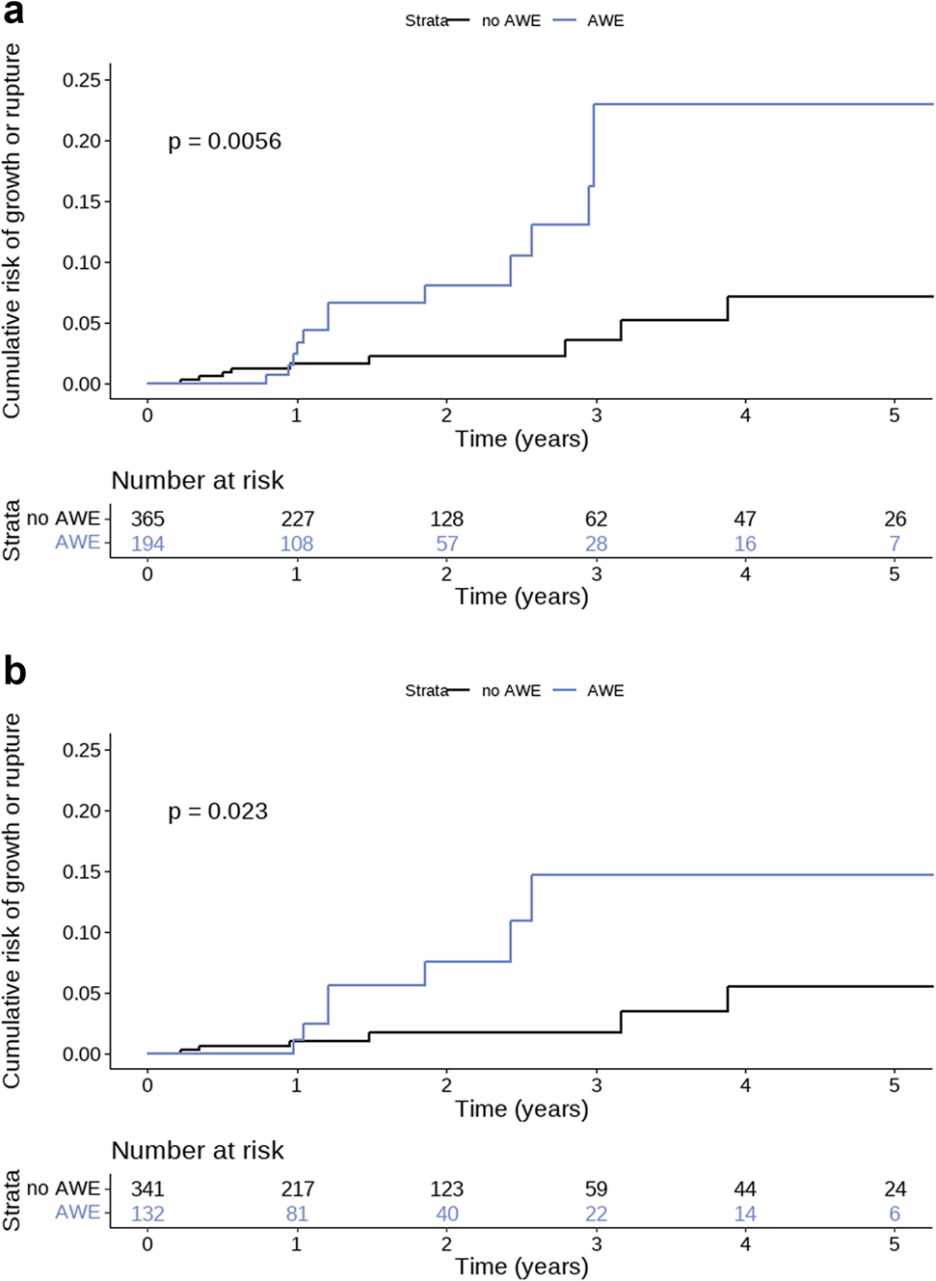
Risk of growth or rupture in aneurysms with and without wall enhancement. **a** Hazard function on aneurysm growth or rupture. **b** Hazard function on aneurysm growth or rupture in a subgroup of aneurysms < 7 mm. Legends: *p* value represents the outcome of the log-rank test. Abbreviations: AWE aneurysm wall enhancement

Aneurysm growth or rupture occurred after a median follow-up of 1.1 years (IQR 0.5–2.1) in 13/194 (6.7%) aneurysms with wall enhancement and after a median follow-up of 1.1 years (IQR 0.7–2.5) in 9/365 (2.5%) aneurysms without wall enhancement (absolute overall risk difference 4.2% [95%CI 0.3–8.1%]). The assumptions of proportional hazard were met. The crude HR of wall enhancement for aneurysm growth or rupture was 3.1 [95%CI 1.3–7.4]. The Kaplan-Meier curve is shown in Figure 1 (log-rank test: *p *= 0.0053). When adjusting for aneurysm size, the HR was 1.4 [95%CI 0.5–3.7] (Table 3). A stratified analysis in male and female patients did not show sex differences in the risk of growth or rupture.

**Table 3:** Cox regression analysis for aneurysm growth or rupture

Analysis	Predictor	Univariable [95%CI]	Bivariable [95%CI]
Main analysis: *n *= 559 aneurysms	Aneurysm wall enhancement	**3.1 [1.3–7.4]**	1.4 [0.5**–**3.7]
	Aneurysm size	**1.4 [1.2–1.5]**	**1.3 [1.2–1.6]**
Subgroup analysis: small (< 7 mm) aneurysms; *n *= 473 aneurysms	Aneurysm wall enhancement	**3.3 [1.1–9.7]**	2.7 [0.8**–**8.9]
	Aneurysm size	2.5 [0.9**–**2.0]	1.2 [0.8**–**1.8]
Subgroup analysis: 3T field strength; *n *= 443 aneurysm	Aneurysm wall enhancement	2.5 [0.9**–**6.8]	1.2 [0.4**–**4.1]
	Aneurysm size	**1.3 [1.1–1.5]**	**1.3 [1.1–1.5]**

Bold indicates statistical significance at an alpha level of 0.05

### Subgroup analysis for aneurysms < 7 mm

In this subgroup analysis, 473 aneurysms with a diameter < 7 mm were included. The median follow-up duration was 1.3 years (IQR 0.7–2.6). Aneurysm growth occurred in 11 (2.3%) aneurysms and rupture occurred in 2 (0.4%) aneurysms during follow-up. Aneurysm growth or rupture occurred in 7/132 (5.3%) aneurysms with wall enhancement and in 6/341 (1.8%) aneurysms without wall enhancement (absolute overall risk difference 3.5% [95%CI−0.5–7.6%]). The HR of aneurysm wall enhancement in this subgroup was 3.3 (95% CI 1.1–9.9). The adjusted HR of aneurysm wall enhancement for risk of aneurysm growth or rupture was 2.7 (95%CI 0.8–8.5). The Kaplan-Meier curve is shown in Figure 1 (log-rank test: *p *= 0.025).

### Subgroup analysis for patients scanned on 3T field strength

In this subgroup analysis, 443 aneurysms were included. The median follow-up duration was 1.2 years (IQR 0.7–2.5). Aneurysm growth occurred in 13 (2.9%) aneurysms and rupture occurred in 2 (0.5%) aneurysms during follow-up. Aneurysm growth or rupture occurred in 8/161 (5.0%) aneurysms with wall enhancement and in 7/282 (2.4%) aneurysms without wall enhancement (absolute overall risk difference 3.1% [95%CI −0.9–7.0%]). The crude HR was 2.5 (95%CI 0.9–6.8) and the adjusted HR of aneurysm wall enhancement for risk of aneurysm growth or rupture was 1.2 (95%CI 0.4–4.1).

## Discussion

This study shows that in patients with an unruptured intracranial aneurysm, gadolinium enhancement is a predictor for growth or rupture during short-term follow-up, but not independent of aneurysm size. Larger aneurysms more often have enhancement of the aneurysm wall and have a higher risk of future aneurysm growth or rupture than smaller aneurysms.

Although many previous studies investigated gadolinium-enhanced aneurysm wall imaging, only three studies had a longitudinal design [15, 25, 26]. In the first study, 57 patients with 65 aneurysms were included [26]. During a median follow-up of 27 months, aneurysm growth or rupture was observed in 4/19 (21%) aneurysms with wall enhancement and 0/46 without wall enhancement. The risk difference of growth and rupture between aneurysms with and without enhancement was 21% (95%CI 3–39%), but no adjustment for aneurysm size was made. In the second study, nine aneurysms were longitudinally followed, of which two (22%) ruptured within 1 year of follow-up [15]. One of the two ruptured aneurysms had wall enhancement at baseline. It was found that the contrast extravasation rate, indicating a higher permeability of the aneurysm wall, was associated with a higher risk of aneurysm rupture. Since no data were given on aneurysm wall enhancement of the other seven longitudinally followed aneurysms, it was not possible to assess whether enhancement was a predictor for growth or rupture. In the most recent study, 129 patients with 145 UIAs were included [25]. During a median follow-up of 24 months, aneurysm growth or morphological change, but not rupture, occurred in 10/65 (15%) aneurysms with wall enhancement at baseline, and in 2/80 (2.5%) aneurysms without enhancement. Presented relative risks were not adjusted for aneurysm size.

Our data suggest that gadolinium enhancement of the aneurysm wall may serve as an additional predictor in the subgroup of aneurysms < 7 mm, because in this subgroup the HR was statistically significant in univariate analysis and the point estimate of the HR only slightly decreased when adjusting for aneurysm size, with a 95% confidence interval slightly crossing 1. It may be that with a larger sample size or a longer follow-up duration, the confidence interval becomes smaller resulting in a statistically significant difference. This subgroup of small aneurysms is the most important to find additional predictors for, because this is the largest group of patients with unruptured aneurysms, and larger aneurysms are usually preventively treated. Especially in this group, it is clinically relevant to find predictors that add value to conventional predictors, which can then be used to improve existing risk prediction models for rupture and growth of unruptured intracranial aneurysms.

A strength of this study is that it is the largest longitudinal aneurysm wall imaging study to date. Previous longitudinal studies did not adjust for aneurysm size. Due to the inclusion of individual patient data of twelve published and unpublished cohorts from three different continents, we had sufficient patient numbers with aneurysm growth or rupture to adjust for the confounder aneurysm size. In addition, several cross-sectional studies used the PHASES score as a surrogate to assess the risk of rupture, since follow-up data were lacking. The current study overcomes these limitations of previous studies, since all aneurysms were followed over time. Furthermore, the radiologists assessed the aneurysm wall, size, and shape prospectively and were therefore blinded for future aneurysm growth or rupture, our primary outcome. Moreover, we used pre-defined inclusion criteria and uniform definitions from the NIH Common Data Elements project on Unruptured Intracranial Aneurysms and Subarachnoid Hemorrhage [28]. This approach added to the generalizability of the study results.

We also need to address a few limitations. First, selection bias inevitably occurred, since only patients who had no preventive aneurysm treatment and had radiological follow-up could be included. This selection of conservatively treated UIA patients might differ between cohorts, since the decision on whether follow-up imaging is advised could differ between centres and countries. However, this selection did not affect our implications, because patients for whom it is decided that follow-up imaging is advised are the targeted group to consider for aneurysm wall imaging. The inclusion of 12 cohorts of patients from different centres adds to the generalizability of the results. Second, our sample size was powered to adjust for one variable and not for other determinants of aneurysm wall enhancement, such as aneurysm shape. However, aneurysm size has been shown to be the strongest determinant of aneurysm wall enhancement [16], thus most important to adjust for. In addition, we had a lower proportion of aneurysms with growth or rupture during follow-up than expected beforehand, which could be explained by a short follow-up period and selection of low-risk aneurysms. Due to the limited number of patients with long-term follow-up and the overall median follow-up period of 1.2 years, our findings cannot be extrapolated to assess the predictive value of aneurysm wall enhancement in the long term. The selection of low-risk aneurysms is reflected by relatively small numbers of patients with an aneurysm at the anterior cerebral artery, posterior communicating artery, or vertebrobasilar arteries. Third, we might not have captured all outcomes since rupture can result in sudden out-of-hospital death, possibly misclassified as sudden cardiac arrest. This could underestimate (if those who died outside the study had aneurysm wall enhancement), overestimate (if those who died outside the study had no wall enhancement), or not affect (in case of an equal distribution of out-of-hospital death from aneurysm rupture in patients with and without aneurysm wall enhancement) the HR in the univariate analysis. The HR when adjusting for size would likely not change, as aneurysm size is the strongest predictor of rupture, and the association between wall enhancement and size holds. Thus, our main outcome is most likely not affected by missing patients with out-of-hospital death who were misclassified as sudden cardiac arrest. Fourth, due to a lack of international consensus on the imaging protocol for aneurysm wall imaging, the imaging protocols differed between sites. This variation in imaging parameters may have affected the visualisation of wall enhancement and the presence of flow artefacts mimicking wall enhancement. We could not include the site as a random effect in our analysis since the number of patients was too small in most of the 12 sites. In addition, the presence of wall enhancement for each aneurysm was not centrally assessed but determined based on a local MR protocol by a local neuroradiologist. This may have resulted in interobserver variability, yet previous studies showed that the interobserver variability for qualitative assessment of wall enhancement is negligible [21, 29].

## Conclusions

Although gadolinium enhancement of the aneurysm wall is a predictor for aneurysm growth and rupture during short-term follow-up, this association was not independent of aneurysm size. As such, aneurysm wall enhancement appears to have no additional value to conventional predictors. Future studies with a longer follow-up period are needed, especially in the subgroup of aneurysms < 7 mm, in order to obtain more precise risk estimates.

### Supplementary Information

Below is the link to the electronic supplementary material.

Supplementary file1 (PDF 205 KB)

## Abbreviations

Acom: Anterior communicating artery
AWE: Aneurysm wall enhancement
HR: Hazard ratio
ICA: Internal carotid artery
IQR: Interquartile range
MCA: Middle cerebral artery
Pcom: Posterior communicating artery
SAH: Subarachnoid haemorrhage
UIA: Unruptured intracranial aneurysm

## Declarations

### Guarantor

The scientific guarantor of this publication is Mervyn D. I. Vergouwen.

### Statistics and biometry

Two of the authors, Laura T. van der Kamp and Irene C. van der Schaaf, have significant statistical expertise.

### Study subjects or cohorts overlap

Some study subjects or cohorts have been previously reported in Stroke 2020 (129 patients), AJNR 2018 (57 patients), and AJNR 2019 (29 patients). We included data of 455 patients from 12 centres. In total, data of 176 patients from 3 centres were previously published. This is outlined in eTable 1, which also includes references to the papers of the 3 monocentre studies. The current study differs from the 3 previous studies: first, this was a multicentre study instead of a monocentre study. Second, we used uniform definitions across all participating centres. Third, we updated the available data which increased the follow-up duration of the previously published patients.

### Methodology

• retrospective

• observational

• multicentre study
