# Supplementary material for: Gadolinium-enhanced intracranial aneurysm wall imaging and risk of aneurysm growth and rupture: a multicentre longitudinal cohort study
Source: Eur Radiol. 2023 Dec 18;34(7):4610–8. doi: 10.1007/s00330-023-10388-7 (PMC11213723; doi:10.1007/s00330-023-10388-7)
Supplement: Supplementary file 1 — Supplementary file1 (PDF 205 KB) [file 330_2023_10388_MOESM1_ESM.pdf]

eTable 1

Cohort	**Included patients N**	**Included aneurysms N**
**Paris, France†**		
Centre Hospitalier Sainte-Anne	125	142
**Hiroshima, Japan**		
Hiroshima City Asa Citizens Hospital	89	115
**Southampton, United Kingdom**		
University Hospital Southampton NHS Foundation Trust	67	98
**Utrecht, The Netherlands‡**		
University Medical Centre Utrecht	76	87
**Washington, United States of America**		
University of Washington School of Medicine	18	26
**San Francisco, United States of America**		
University of California San Francisco	23	26
**Bejing, China**		
Capital Medical University Xuanwu Hospital	21	26
Tsinghua University[c]	3	3
**Hiroshima, Japan**		
Hiroshima University	9	12
**Shanghai, China**		
Changhai Hospital	11	11
Ren Ji Hospital	3	3
**Zhengzhou, China**		
The First Affiliated Hospital of Zhengzhou University	10	10

† 125 patients from Paris were previously described in the study by Gariel et al (Stroke 2020; 129 patients). Patients with >15 mm were excluded for the current study. Moreover, morphological change was not considered as an outcome event in the current study.

‡ 48 patients from Utrecht were previously described in the study by Vergouwen et al (AJNR 2019; 57 patients). Patients with CTA follow-up were excluded for the current study.

§ 3 patients from Bejing were previously described in the study by Qi et al (AJNR 2019; 29 patients). Patients without follow-up were excluded for the current study.

eTable 2 Imaging parameters of the cohorts (1/2)

**Cohort**	**Centre Hospitalier Sainte-Anne**	**Hiroshima City Asa Citizens Hospital**	**University Hospital Southampton NHS Foundation Trust**	**University Medical Centre Utrecht**			**University of Washington School of Medicine**
**Vendor**	GE Healthcare	GE Healthcare	Siemens	Philips	Philips	Philips	Siemens
**Magnet strength**	3T	1.5T	3T	3T	3T	7T	3T
**Acquired spatial resolution (mm)**	0.9×0.9×1	1x1x1	0.5x0.5x0.5	0.5 x 0.5 x 1	0.6 x 0.6 x 1	0.6x0.6x0.6	0.56x0.56x0.56
**Reconstructed spatial resolution (mm)**	0.45×0.45×0.5	1x1x1	0.5x0.5x0.5	0.5x0.5x0.5	0.5 x 0.5 x 0.5	0.6x0.6x0.6	0.56x0.56x0.56
**Time between gd injection and start AWI gd (min)**	1	1:30	2	5	5	4:30	5
**Acquisition time of vessel wall imaging (min)**	4:16	3:39	8	8:03	6	8:03	8:08 (T1 SPACE); 5:11 (DANTE CAIPI)
**Pulse sequence type**	3DT1- CUBE FastSpinEcho with variable refocussing flip angle	3D FSE CUBE	3DT1- FastSpinEcho with variable flip angle train	3D multishot turbo spin-echo sequence	3D multishot turbo spin-echo sequence	Turbo field echo	3DT1 SPACE with variable refocussing flip angle; 3D turbo spin-echo sequence
**Repetition time (ms)**<br>**Echo time (ms)**	600<br>11.5	550<br>10	938<br>24	1.500<br>36	1500<br>32.4	8.2<br>4.4	1100<br>11
**Flow-suppression method**	Black blood	Black blood	Black blood	Black blood Anti drive pulse with tissue specific refocussing control	Black blood Anti drive pulse with tissue specific refocussing control	Black blood	non-DANTE T1 space; DANTE
**Neuro-radiologists' experience**	≥15 years	>25 years	>20 years	>10 years	>15 years	>10 years	≥19 years

Abbreviations: gd = gadolinium; AWI = aneurysm wall imaging; DANTE = Delay Alternating with Nutation for Tailored Excitation.

eTable 2	Imaging parameters of the cohorts (2/2)						
**Cohort**	**University of California San Francisco**	**Capital Medical University Xuanwu Hospital**	**Tsinghua University**	**Hiroshima University**	**Changhai Hospital**	**Ren Ji Hospital**	**The First Affiliated Hospital of Zhengzhou University**
**Vendor**	Siemens	Siemens	Philips	Philips	Siemens	Siemens	Siemens
**Magnet strength**	3T	3T	3T	3T	3T	3T	3T
**Acquired spatial resolution (mm)**	0.5x0.5x0.5	0.6x0.6x0.7	0.6x0.6x0.6	0.98x 0.99 x 1	0.5x0.5x0.5	0.6x0.6x0.6	0.6x0.6x0.6
**Reconstructed spatial resolution (mm)**	0.5x0.5x0.5	0.6x0.6x0.7	0.6x0.6x0.6	0.46x0.46 x0.5	0.5x0.5x0.5	0.6x0.6x0.6	0.6x0.6x0.6
**Time between gd injection and start AWI gd (min)**	2	2	6	5	2	2	2
**Acquisition time of vessel wall imaging (min)**	9:58	8:14	4:23	4:42	8	9:13	7:36
**Pulse sequence type**	3D SPACE (turbo spin echo with variable flip angle train)	3D SPACE (turbo spin echo with variable flip angle train)	3D VISTA (turbo spin echo with variable flip angle train)	3D multishot turbo spin-echo sequence	3D SPACE (turbo spin echo with variable flip angle train)	3D SPACE (turbo spin echo with variable flip angle train)	3D SPACE (turbo spin echo with variable flip angle train)
**Repetition time (ms)<br>Echo time (ms)**	1000<br>17	900<br>20	700<br>30	400<br>13	900<br>5.6	1000<br>15	800<br>14
**Flow-suppression method**	Black blood	Black blood	Black blood	Black blood, MSDE	Black blood	Black blood	Black blood
**Neuro-radiologists' experience**	>10 years	>10 years	>10 years	>10 years	>10 years	>10 years	>10 years

Abbreviations: gd = gadolinium; AWI = aneurysm wall imaging; MSDE = motion-sensitized driven-equilibrium
